# Supplementary material for: Investigation of mitochondrial phenotypes in motor neurons derived by direct conversion of fibroblasts from familial ALS subjects
Source: Cell Death Dis. 2025 Nov 21;17(1):51. doi: 10.1038/s41419-025-08126-6 (PMC12811294; doi:10.1038/s41419-025-08126-6)
Supplement: Supplementary file 1 — Supplementary legends [file 41419_2025_8126_MOESM1_ESM.docx]

**Supplemenrary figure legends**

Supplementary Figure 1. Performance of the ResNet-50 architecture machine learning model for iMN classification. (A) Accuracy plot showing training (blue) and test (red) accuracies over 10 epochs for the model used to classify cells as dead, non-neuron, or iMN. (B) Receiver Operating Characteristic (ROC) curve with Area Under the Curve (AUC) values for each class on the holdout test set, demonstrating the model’s performance in distinguishing between dead, non-neuron, and iMN classes. (C) Confusion matrix for the classification model on the holdout test set, showing the true and predicted labels for each category. (D) Normalized confusion matrix, displaying the fraction of correctly and incorrectly classified labels for dead, non-neuron, and iMN categories. (E-G) Example images of cells in each classification category—dead, non-neuron, and iMN—from three different staining conditions: hSyn-eGFP live imaging (E), TDP-43 immunocytochemistry (F), and TMRM live imaging (G). Red arrowheads indicate the cell (or debree) in the exact center which is being classified.

Supplementary Figure 2. Representative images of seahorse experiments.

Supplementary Figure 3. Training and validation metrics, model precision-recall, and regression analyses for iMN classification and survival analysis. (A) Training and validation loss curves for box loss, classification loss, distance focal loss (dfl), Mean Average Precision (mAP) across different Intersection over Union (IoU) thresholds for bounding box detection, and Precision-recall curve, with the average precision across all classes and specific mAP scores at different IoU thresholds during model training, showing the convergence of these metrics over 6000 steps. Precision - recall metrics for the model on the validation set (B).

Supplementary Figure 4. iMN survival half-life (A,B,C) and transformation efficiency (D, E, F) condounded by passage (A, D), age at biopsy (B, E), and sex (C, F), showing R-squared values and p-values for each relationship, suggesting no confounding effects in our data.

Supplementary Figure 5. Summary of key bioenergetic findings from iMN analyses across control and fALS genotypes. Heatmap displaying percent changes for multiple mitochondrial and bioenergetic parameters in iMN derived from fALS versus control iMN. Percent changes are calculated relative to control iMN, with minus log2-transformed p-values indicated in parentheses. Only parameters with statisticaly significant differences are shown.
