# Supplementary figures and images for: Investigation of mitochondrial phenotypes in motor neurons derived by direct conversion of fibroblasts from familial ALS subjects
Source: Cell Death Dis. 2025 Nov 21;17(1):51. doi: 10.1038/s41419-025-08126-6 (PMC12811294; doi:10.1038/s41419-025-08126-6)

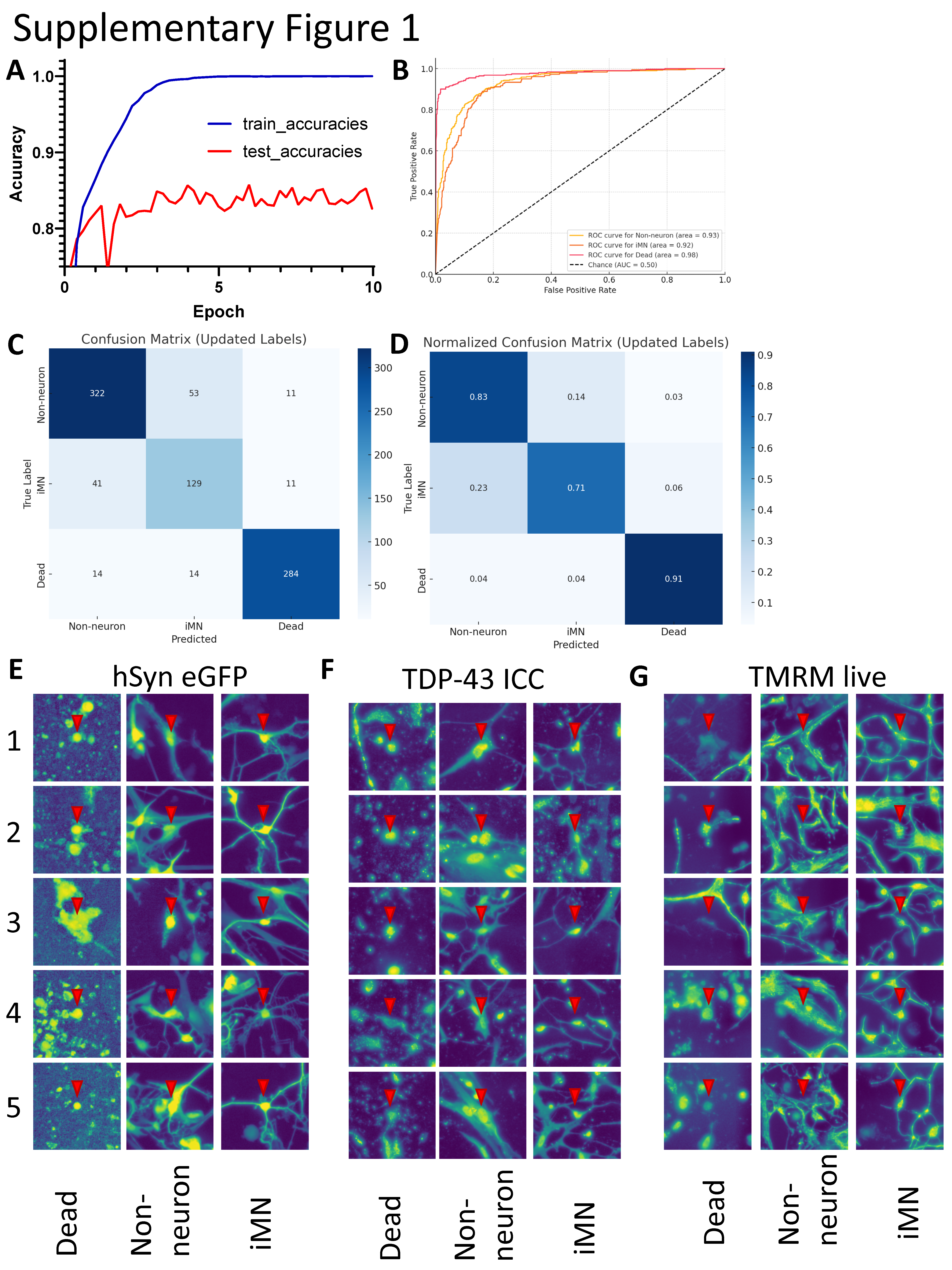

Supplement: Supplementary file 2 — Supplementary Figure 01 [file 41419_2025_8126_MOESM2_ESM.tif]

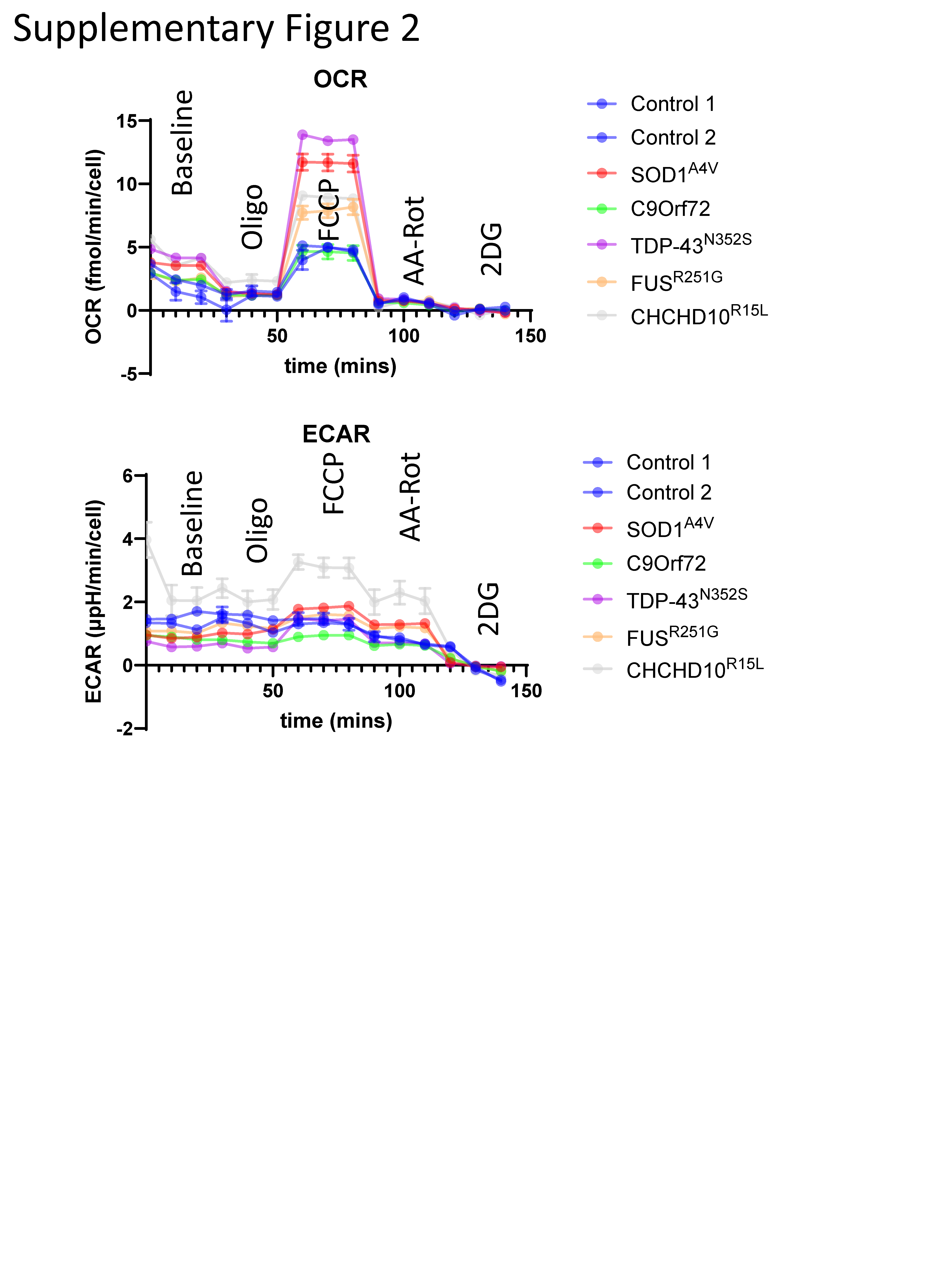

Supplement: Supplementary file 3 — Supplementary Figure 02 [file 41419_2025_8126_MOESM3_ESM.tif]

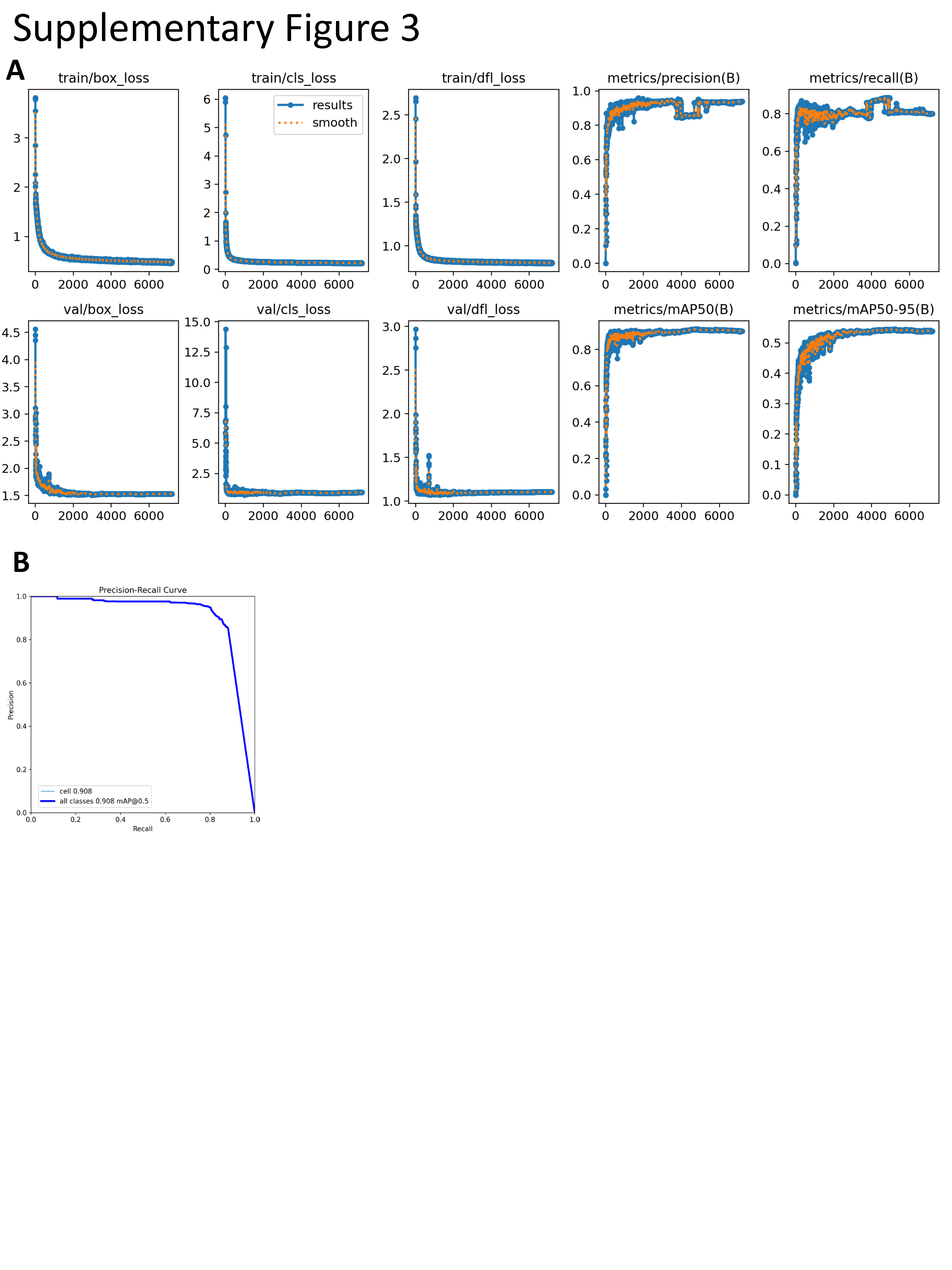

Supplement: Supplementary file 4 — Supplementary Figure 03 [file 41419_2025_8126_MOESM4_ESM.tif]

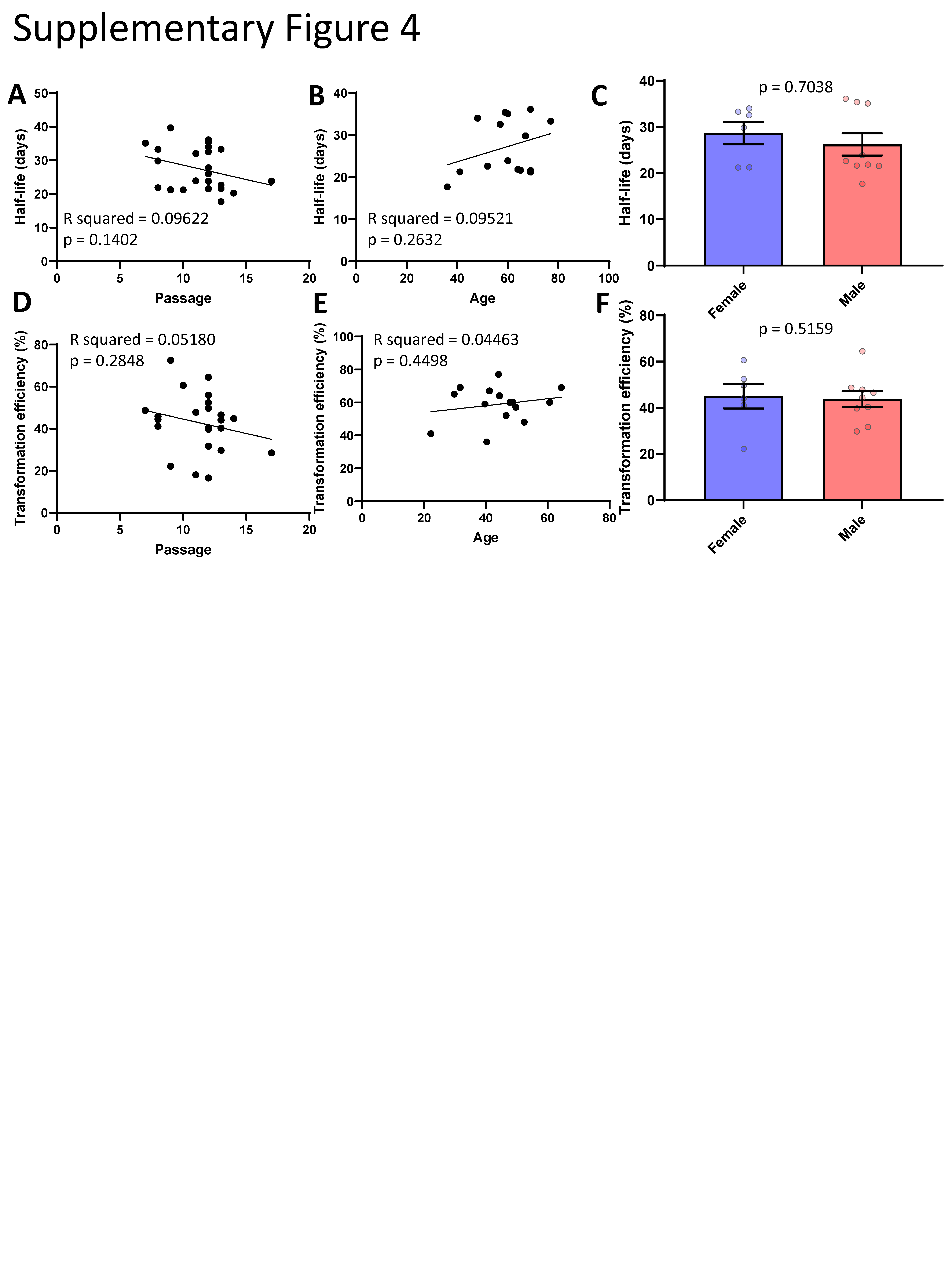

Supplement: Supplementary file 5 — Supplementary Figure 04 [file 41419_2025_8126_MOESM5_ESM.tif]

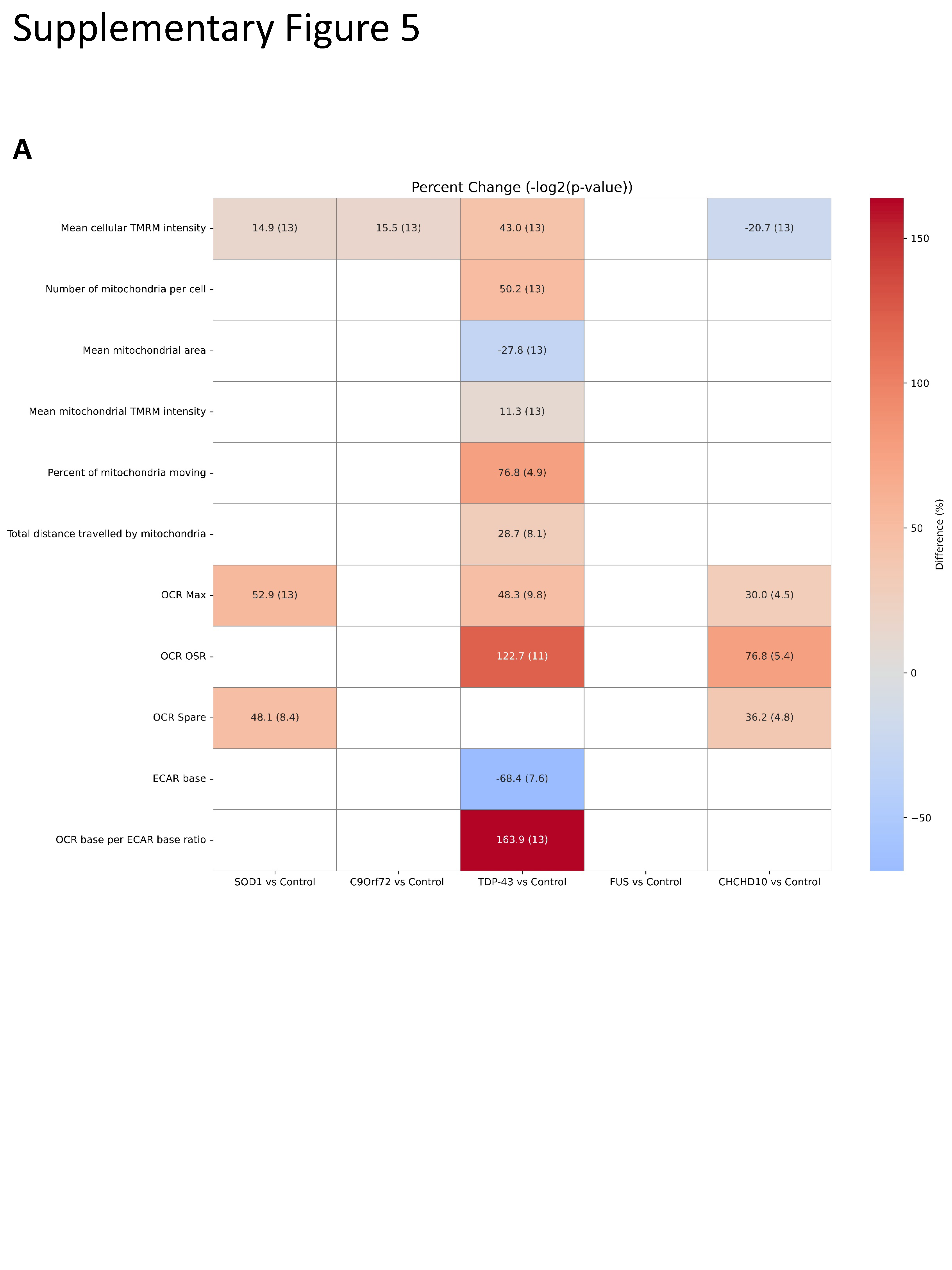

Supplement: Supplementary file 6 — Supplementary Figure 05 [file 41419_2025_8126_MOESM6_ESM.tif]
